# Intracranial aneurysm instability prediction model based on 4D-Flow MRI and HR-MRI

**DOI:** 10.1016/j.neurot.2024.e00505

**Published:** 2024-11-30

**Authors:** Fei Peng, Jiaxiang Xia, Fandong Zhang, Shiyu Lu, Hao Wang, Jiashu Li, Xinmin Liu, Yao Zhong, Jiahuan Guo, Yonghong Duan, Binbin Sui, Chuyang Ye, Yi Ju, Shuai Kang, Yizhou Yu, Xin Feng, Xingquan Zhao, Rui Li, Aihua Liu

**Affiliations:** a Beijing Neurosurgical Institute and Beijing Tiantan Hospital, Capital Medical University, Beijing, China; b Deepwise Artificial Intelligence (AI) Lab, Deepwise Inc., Beijing, China; c School of Integrated Circuits and Electronics, Beijing Institute of Technology, Beijing, China; d Department of Neurology, Beijing Tiantan Hospital, Capital Medical University, Beijing, China; e Department of Neurosurgery, The Second Affiliated Hospital, Hengyang Medical School, University of South China, Hengyang, Hunan, China; f Tiantan Neuroimaging Center of Excellence, China National Clinical Research Center for Neurological Diseases, Beijing, China; g Department of Neurosurgery, Beijing Tiantan Hospital, Capital Medical University, Beijing, China; h Department of Computer Science, The University of Hong Kong, Hong Kong, China; i Neurosurgery Center, Department of Cerebrovascular Surgery, Zhujiang Hospital, Southern Medical University, Guangzhou, Guangdong, China; j Center for Biomedical Imaging Research, Department of Biomedical Engineering, School of Medicine, Tsinghua University, Beijing, China

**Keywords:** Intracranial aneurysm, MRI, Machine learning, Ensemble learning, Hemodynamics

## Abstract

This study aims to develop a reliable predictive model for assessing intracranial aneurysm (IA) instability by utilizing four-dimensional flow magnetic resonance imaging (4D-Flow MRI) and high-resolution MRI (HR-MRI). Initially, we curated a prospective dataset, dubbed the primary cohort, by aggregating patient data that was consecutively enrolled across two centers from November 2018 to November 2021. Unstable aneurysms were defined as those with symptoms, morphological change or ruptured during follow-up periods. We introduce a specialized ensemble learning framework, termed the Hybrid Model, which synergistically combines two heterogeneous base learning algorithms: 4D-Flow logistic regression (4D-Flow-LR) and Multi-crop Attention Branch Network (MicroAB-Net). The ability of the hybrid model to predict aneurysm instability was compared with baseline models: PHASES (population, hypertension, age, size, earlier rupture, and site) LR, ELAPSS (earlier subarachnoid hemorrhage, location, age, population, size, and shape) LR, aneurysm wall enhancement (AWE) LR, and Radiomics using the area under the curve (AUC) with Delong's test. Finally, the Hybrid Model was further validated in the validation cohort (patients enrolled between December 2021 to May 2022). In the primary cohort, 189 patients (144 women [76.2 ​%]; aged 58.90 years ​± ​10.32) with 213 IAs were included. In the validation cohort, 48 patients (35 women [72.9 ​%]; aged 55.0 years ​± ​10.77) with 53 IAs were included. The Hybrid Model achieved the highest performance both in the primary cohort (AUC ​= ​0.854) and the validation cohort (AUC ​= ​0.876). The Hybrid model provided a promising prediction of aneurysm instability.

## Introduction

Intracranial aneurysms (IAs) are present in nearly 3 ​% of the global population [1]. Although the annual rupture risk is as low as 0.95 ​%, once an aneurysm ruptures, it creates a catastrophic event with high mortality (nearly 50 ​%) and high morbidity (nearly 20 ​%) [2]. Furthermore, preventive treatment of unruptured IAs is also accompanied by the risk of procedure-related complications (4.96 ​%) [3]. Therefore, timely and accurate identification of unstable IAs (i.e. with a high risk of growth or rupture) is of paramount importance in determining the optimal opportunity for surgical intervention and optimal therapeutic modalities [4].

Previous models based on large cohort studies for predicting unstable IAs included PHASES (population, hypertension, age, size, earlier rupture, and site) score, and ELAPSS (earlier subarachnoid hemorrhage, location, age, population, size, and shape) score [5,6]. Recently, more and more studies revealed that both aberrant intra-aneurysmal blood flow and vulnerable aneurysm wall contribute to intracranial aneurysm growth or rupture [[7], [8], [9]] However, most of the previous prediction models were based on computational fluid dynamics (CFD) and lumen-based cerebral angiography [10,11]. They may fail to obtain the real intra-aneurysmal hemodynamics and the detailed characterization of the intracranial aneurysmal wall. Four-dimensional flow magnetic resonance imaging (4D-Flow MRI) is an emerging technique for evaluating hemodynamics that provides real-time and reliable blood flow and velocity data, which has been used in risk stratification of IAs [12]. As per the aneurysmal wall, high-resolution MRI (HR-MRI) provides valuable information (i.e. aneurysm wall enhancement [AWE], thickness, components) and insights into specific pathological processes of the aneurysm wall [13], and AWE has been reported to be a biomarker of aneurysmal wall inflammation and unstable IAs [14,15]. Since recent studies revealed that aneurysmal wall inflammation plays a key role in aneurysmogenesis, progression, and rupture [8], several qualitative (AWE type classification) and quantitative (contrast ratio of the signal intensity between the aneurysmal wall and the pituitary stalk, CR_stalk_) analyses of AWE have been performed to confirm the predictive value of AWE in IA instability prediction [14,15]. However, the use of AWE type classifications and quantitative parameters as proxies for detailed aneurysmal wall information may be limited because AWE type assessment is subjective, and the quantitative parameters only just summarize the information contained in the HR-MRI into a single value.

Recently, machine learning (ML) has become increasingly used in IA detection, instability or rupture prediction [10,11,16]. ML enables learning and training models to collect and remember features and parameters, which has shown incremental value [17]. Zhu et al. combined clinical and morphological features of IAs by using ML models, and the results revealed a superior performance of the ML models compared with previous models [18]. Deep learning, a subset of machine learning, uses deep neural networks that automatically learn to extract features from images, and incorporates features of biochemical and mechanical processes into a data-driven model [17,19]. We hypothesized that some detailed information present in HR-MRI is not identified by readers or captured by simple contrast-ratio measurements, and that deep learning would leverage such information and generate improved risk models.

In this study, we aimed to propose a novel hybrid model (machine learning model based on 4D-Flow MRI and deep learning model based on HR-MRI) for predicting aneurysm instability to facilitate clinicians in decision-making on surgical treatment. To test the IA prediction ability, we compared our model with several previous models that have been reported previously (PHASES score, ELAPSS score, AWE, Radiomics, etc.) [5,6,11,14,15]. Then, our model was further validated in the validation cohort.

## Methods

### Study design and participants

The current study was extended by a multicenter prospective Chinese Clinical Trial Registry to study the associations between aneurysm risk factors based on 4D-Flow MRI and HR-MRI. Previously described case series and cohort studies have demonstrated the effectiveness and quality for this research [9,20,21]. The current study was approved by the local institutional review board (IRB approval number: KY 2018-086-03) and conducted under the guidance of the 1964 Declaration of Helsinki. Written informed consents were obtained from each subject.

**Fig. 1:**
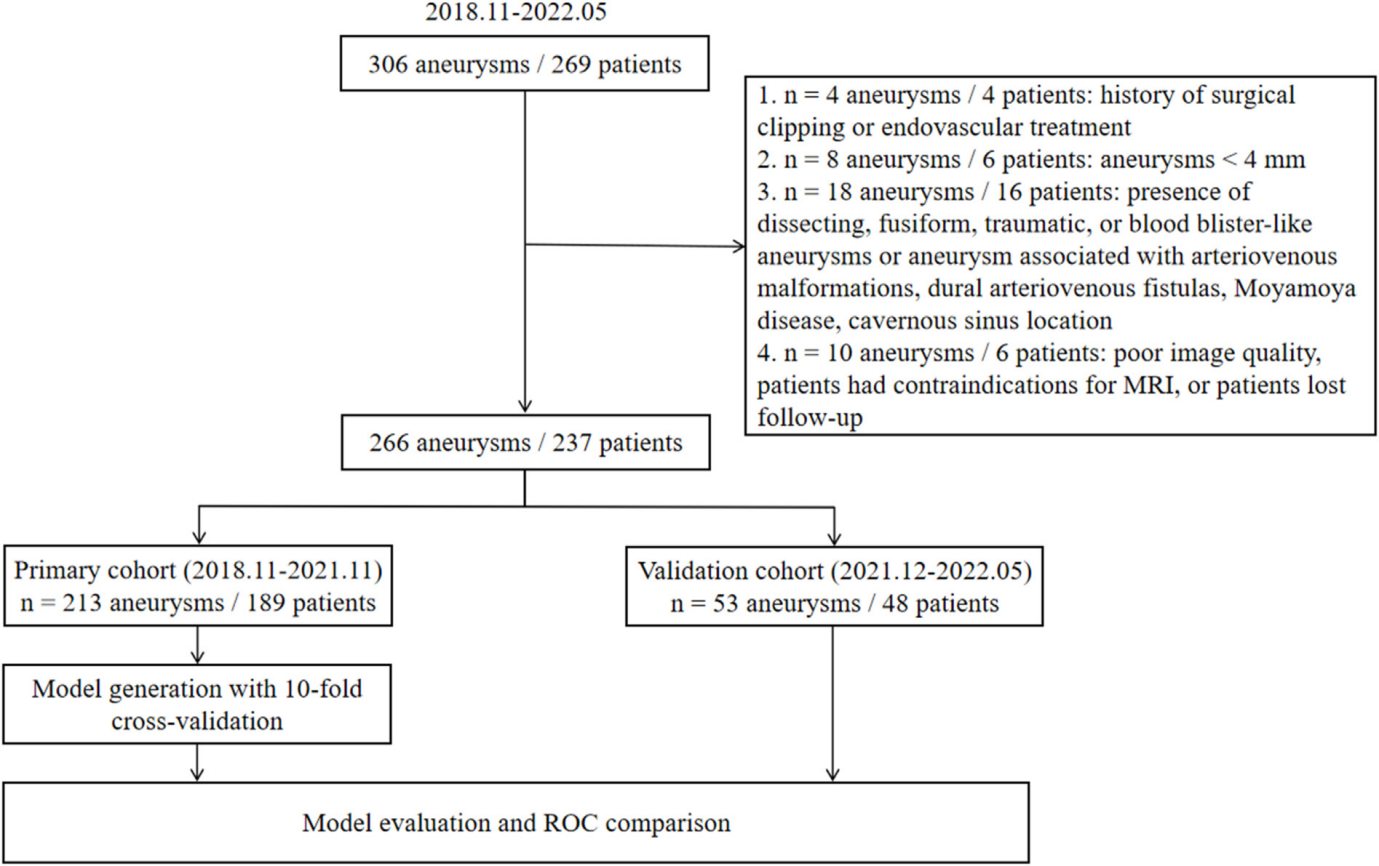
**Patients inflow chart**. MRI, magnetic resonance imaging; ROC, receiver operating characteristic curve.

We reviewed our prospective collected database for consecutive patients with unruptured intracranial aneurysms recruited from November 2018 to May 2022 ​at two centers (269 patients with 306 IAs). Among them, those recruited between December 2018 to December 2021 were included in the primary cohort, and those recruited between November 2021 and May 2022 were included in the validation cohort (Fig. 1). Patients with no symptoms were followed up with imaging after one year to find aneurysm growth or rupture. The patient characteristics recorded included age, sex, hypertension, diabetes, dislipidemia, coronary artery disease, smoking history, PHASES (population, hypertension, age, size, earlier subarachnoid hemorrhage, and site) score [5], and ELAPSS (earlier subarachnoid hemorrhage, location, age, population, size, and shape) score [6]. The exclusion criteria were age <18 years; history of surgical or endovascular treatment; IAs <4 ​mm; presence of dissecting, fusiform, traumatic, or blood blister-like aneurysms or aneurysm associated with arteriovenous malformations, dural arteriovenous fistulas, or Moyamoya disease; history of recent rupture; patients lost to follow-up; patients who had contraindications for MRI; and poor MRI image quality.

### Aneurysm status definition

Aneurysm status was classified as stable or unstable, and the status was evaluated per aneurysm rather than per patient. Unstable IAs were defined as IAs ruptured during 1-year of follow-up, presented with noted morphological change (occurrence of a bleb, regular to irregular, or noted growth >1 ​mm) compared with previous imaging or during 1-year of follow-up, or those in patients with aneurysmal symptoms (thunderclap headache or oculomotor nerve palsy) [15]. Stable IAs were defined as asymptomatic aneurysms without morphological change during 1-year of follow-up [15].

### Imaging protocol

All MRI scan were performed on a 3.0T MRI scanner (Philips Achieva, Best, Netherlands) with a 32-channel head coil. Scan parameters were: TR/TE ​= ​8.0/3.6 ​ms, FOV ​= ​160 ​× ​160 ​× ​30 ​mm^3^, voxel size ​= ​1 ​× ​1 ​× ​1 ​mm^3^, VENC ​= ​120 ​cm/s. The IA walls were imaged using a 3D black-blood T1-weighted volumetric isotropic turbo spin echo acquisition (T1-VISTA) sequence, The imaging parameters were as follows: TR/TE ​= ​800/21 ​ms, FOV ​= ​200 ​× ​180 ​× ​40 ​mm^3^, voxel size ​= ​0.6 ​× ​0.6 ​× ​0.6 ​mm^3^. Post-contrast T1W-VISTA was performed about 6 ​min after an intravenous injection of GdDTPA (Magnevist; Bayer Schering Pharma, Berlin, Germany) at a dose of 0.1 ​mmol/kg. All imaging parameters were kept the same for the pre- and post-contrast-enhanced T1W-VISTA imaging.

### Aneurysm wall enhancement assessment

For the qualitative analysis of the post-contrast T1W-VISTA images, 2 experienced neuroradiologists (20 and 15 years of experience in neuroradiology, respectively) who were blinded to patients’ information and aneurysm status reviewed the post-contrast T1W-VISTA images independently and determined the types of AWE. Any discrepancy would be resolved by consensus. According to previous studies, aneurysm wall enhancement has been classified into four types: grade 0, no enhancement; grade 1, focal enhancement; grade 2, circumferential enhancement; grade 3, thick circumferential enhancement (>1 ​mm) [15]. Discordances between them were resolved by consensus.

CR_stalk_, defined as the contrast ratio of the average signal intensity of the aneurysm wall to the average value of four randomized points in the pituitary stalk, has been proved to be a reliable quantitative parameter to predict aneurysm instability [14]. Illustrations of AWE and CR_stalk_ were provided in Figs. S1–2. A senior neuroradiologist defined the four points in the pituitary stalk, and the enhancement of the aneurysm wall was measured by another two experienced readers.

### Hemodynamics assessment

We used 4D-Flow MRI to monitor the hemodynamic status inside the aneurysm. Preprocessing steps including eddy current correction, application of velocity masks, and vessel segmentation were performed before data visualization and quantification. For the hemodynamics assessment, 2 experienced neuroradiologists who were blinded to patients’ information and aneurysm status assessed the hemodynamics independently. Velocity-weighted masks were adopted to segment the IA, and streamlines were provided for the visualization and quantification of blood flow.

The hemodynamic parameters of minimum, average, and maximum through-plane velocity (V_min_, V_avg_, V_max_, respectively) and average and maximum blood flow (Flow_avg_, Flow_max_, respectively) were measured in the contour of the IA at the largest cross-sectional plane containing the maximum velocity vector. The pulsatility index (PI) was calculated following the method of previous studies [22]. Wall shear stress (WSS) was defined as the velocity gradient along the perpendicular direction of the IA wall, and the time-averaged and the maximum WSS were calculated along the same contour in the IA (TAWSS, WSS_max_; respectively). Parameters were measured using GTFlow, version 2.2.15 (GyroTools, Zurich, Switzerland). Streamlines and the hemodynamic parameter measurement process are shown in Fig. S3.

### Development of the hybrid model

**Fig. 2:**
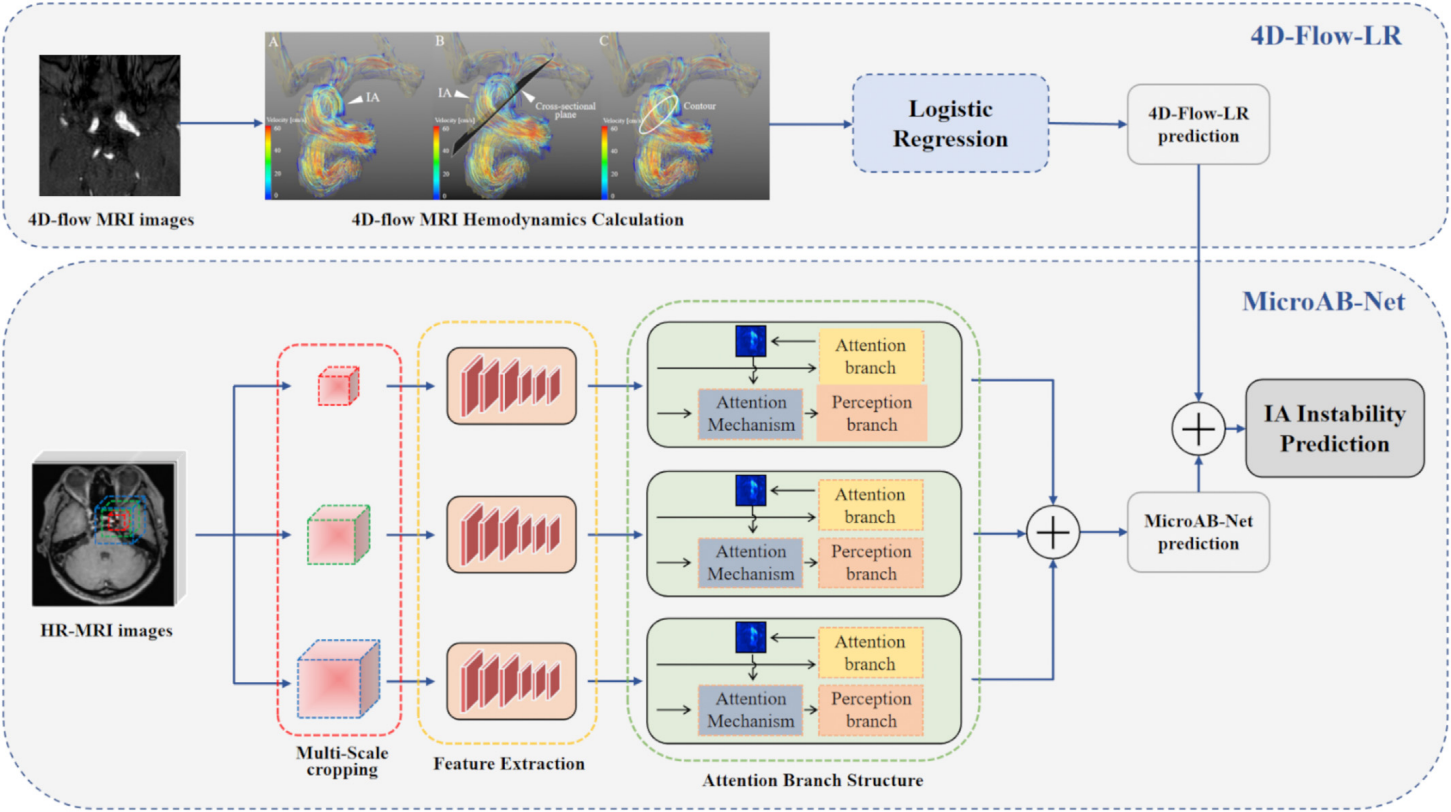
**Structure of the Hybrid Model**. The proposed ensemble learning hybrid model consists of two heterogeneous base learning algorithms. The upper branch of the figure indicates the 4D-Flow-LR model, which is a logistic regression using 4D flow data as input. The lower branch is a novel deep learning model named MicroAB-Net trained by HR-MRI images. The MicroAB-Net contains three main modules: Multi-Scale Cropping, Feature Extraction, and Attention Branch. We fuse the output of both models to generate the final IA instability prediction. HR-MRI, high-resolution magnetic resonance imaging; IA, intracranial aneurysm; LR, logistic regression; MicroAB-Net, Multi-crop Attention Branch Network model.

Our proposed ensemble learning framework, termed the Hybrid Model, contains two heterogeneous base learning algorithms: the 4D-Flow-LR, a machine learning (logistic regression, LR) model that uses 4D-Flow measure information as input; the MicroAB-Net (Multi-crop Attention Branch Network), a deep learning model that takes patches of various times aneurysm lesion size surrounding region in HR-MRI as input, which shown in Fig. 2. For the 4D-Flow-only model, we trained a logistic regression model to map the 4D-Flow hemodynamic parameters of a given aneurysm to its stability status: unstable or not.

To explore both aneurysmal wall and aneurysm morphology-related features, we designed a multiple-crop architecture inspired by previous studies [23]. As can be seen in Fig. 2, given the 3D bounding box of an aneurysm lesion we cropped three different times (2, 3, 4) size surrounding region as patches and fed them into three separate feature extraction networks. All the feature extraction networks had the same structure and shared weights. To make the networks deep and powerful, we introduced residual convolution blocks into the feature extraction networks. Lastly, we applied the attention-branch mechanism to the learned feature maps, being inspired by the work of Fukui et al. [24]. In this way, we could not only boost the representation ability of the model, but also obtain a spatial heatmap of the input aneurysm to better reveal the spatial importance learned by the MicroAB-Net. The attention-branch module consists of a feature extraction module, an attention branch, and a perception branch. The feature extractor contains multiple convolution layers for extracting feature maps. The attention branch is designed to apply an attention mechanism by introducing a response-based visual explanation model that can generate an attention map for the attention mechanism and a visual explanation, as shown in Fig. S4. The perception branch outputs the class probabilities using the feature and attention maps applied to the convolution layers. The detailed network design is shown in the Methods in the **Data Supplement**.

Finally, we built the Hybrid Model by averaging the predicted probabilities of both the MicroAB-Net and the 4D-Flow-LR models.

### Radiomics model details

In the radiomics model, the open source Python library Pyradiomics is used to extract radiomics features of the 3D aneurysm regions. A total of 1906 radiomic features are extracted, which contain first-order statistics, shape (3D), gray-level co-occurrence matrix (GLCM), gray level size zone matrix (GLSZM), gray level run length matrix (GLRLM), gray level dependence matrix (GLDM) and Neighbouring Gray Tone Difference Matrix (NGTDM). Then, 92 radiomic features were selected by the Joint Hypothesis Test to mitigate model overfitting. In detail, if the linear correlation coefficient between any two features is greater than a given threshold (here we use 0.9), the one with less impact on the aneurysm instability classification will be discarded. Last, a logistic regression classification model is performed to map the selected features into aneurysm stability score. In addition, we use L1 regularization during logistic regression to further mitigate overfitting. Details of the radiomics model are provided in the Methods in the **Data Supplement**.

### Development of baseline models

To indicate the effectiveness of our proposed hybrid model, we built several baseline models for comparison. These were a set of logistic regression models using PHASES score (PHASES-LR), ELAPSS score (ELAPSS-LR), AWE method (AWE-LR), and radiomics features (Radiomics) as separate inputs. During training of the above logistic regression models, L1 regularization was used to mitigate overfitting. Finally, the variables included in baseline models are listed in Table S1.

### Statistical analysis

The Shapiro-Wilk test was used to test data normality. SPSS version 26.0 (IBM Corporation, Armonk, NY, USA) was used to perform the statistical analyses of baseline characteristics to identify parameters showing significant differences between stable and unstable IAs. Categorical baseline characteristics were compared using the χ^2^ test and the Mann-Whitney *U* test was used to compare continuous variables. To assess the interobserver reliability of hemodynamic and AWE variables measured by the two readers, intraclass correlation coefficient and Cohen's kappa were used [25].

To evaluate our proposed hybrid model, all models were trained and validated using 10-fold cross-validation performed in Python 3.6. The 10 folds are divided into the patient level, and all the models share the same divide. The evaluation criteria included AUC (area under the receiver operating characteristic), accuracy, sensitivity, specificity, PPV (positive predictive value), and NPV (negative predictive value). The 95 ​% confidence intervals (CI) of the AUCs and the 95 ​% CIs of the proportion-based criteria (accuracy, sensitivity, specificity, PPV, and NPV) were calculated following the methods of previous studies [26,27]. Model performance was compared by using AUCs with the DeLong's test. The level of statistical significance was set at P ​< ​0.05.

## Results

### Demographic and clinical features

Initially, 269 patients with 306 aneurysms were recruited. Among them, 32 patients were excluded: 4 patients with history of surgical clipping or endovascular treatment; 6 patients with IAs< 4 ​mm; 16 patients presented with dissecting, fusiform aneurysms, moyamoya disease or arteriovenous malformations, cavernous sinus location, or with a history of recent rupture; 6 patients with poor MRI image quality or lost follow-up. Finally, 213 IAs were included in the primary cohort, and 53 IAs were included in the validation cohort (Fig. 1). In the primary cohort, three aneurysms manifested with noted morphological change compared with previous examinations, and 48 were symptomatic. Thus, at baseline, 51 aneurysms were classified as unstable IAs. Among the stable aneurysms at baseline, three ruptured and ten manifested noted morphological change during the 1-year follow-up. Therefore, in the primary cohort, 64 aneurysms were finally classified as unstable, while 149 aneurysms were stable. The demographics and clinical features are summarized in Table 1.

**Table 1:** Demographic and clinical characteristics between stable and unstable aneurysms.

Variables	Primary cohort, N ​= ​213			Validation cohort, N ​= ​53		
	Stable IAs, N ​= ​149	Unstable IAs, N ​= ​64	P value	Stable IAs, N ​= ​35	Unstable IAs, N ​= ​18	P value
Age, years	59.82 ​± ​9.12	56.75 ​± ​12.50	0.167	55.14 ​± ​12.16	54.72 ​± ​7.68	0.756
Female, N (%)	101 (67.8)	43 (73.4)	0.516	25 (71.4)	10 (55.6)	0.359
Hypertension, N (%)	103 (69.1)	39 (60.9)	0.269	17 (48.6)	10 (55.6)	0.773
Diabetes, N (%)	13 (8.7)	9 (14.1)	0.325	4 (11.4)	4 (22.2)	0.421
Dislipidemia, N (%)	53 (35.6)	27 (42.2)	0.441	10 (28.6)	5 (27.8)	1.000
Coronary artery disease, N (%)	20 (13.4)	4 (6.3)	0.160	1 (2.9)	2 (11.1)	0.263
Smoking history, N (%)	42 (28.2)	13 (20.3)	0.305	8 (22.9)	6 (33.3)	0.535
Aneurysm location, N (%)			0.607			0.040^a^
Anterior circulation	113 (75.8)	46 (71.9)		26 (74.3)	9 (25.7)	
Posterior circulation	36 (24.2)	18 (28.1)		8 (44.4)	10 (55.6)	
Side-wall, N (%)	53 (35.6)	17 (26.6)	0.208	26 (74.3)	14 (77.8)	1.000
Mural thrombus, N (%)	27 (18.1)	38 (59.4)	<0.001^a^	4 (11.4)	5 (27.8)	0.245
Size	6.91 ​± ​3.50	12.11 ​± ​6.16	<0.001^a^	6.20 ​± ​2.12	8.80 ​± ​2.64	<0.001^a^
Phases Score	5.20 ​± ​3.57	8.56 ​± ​2.78	<0.001^a^	5.71 ​± ​4.16	8.61 ​± ​2.89	0.025^a^
Elapss Score	17.00 ​± ​7.13	23.98 ​± ​4.77	<0.001^a^	19.97 ​± ​7.73	24.83 ​± ​4.64	0.024^a^

a Significant values. IA, intracranial aneurysm.

### Hemodynamic and AWE features

Baseline characteristics of hemodynamic and AWE features were provided in Table 2. The interobserver reliability was excellent between the two readers for the measurement of hemodynamic and AWE variables (Table S2). For the primary cohort, the median CR_stalk_ was 0.55 ​± ​0.17 for stable IAs and 0.72 ​± ​0.22 for unstable IAs (p ​< ​0.001). Both the median PHASES score (5.20 ​± ​3.57 vs 8.56 ​± ​2.78) and Elapss score (17.00 ​± ​7.13 vs 23.98 ​± ​4.77) were significantly higher in unstable IAs compared with stable IAs (both p ​< ​0.001, Table 2).

**Table 2:** Aneurysm wall enhancement and hemodynamic characteristics of all intracranial aneurysms.

Variable	Primary cohort, N ​= ​213			Validation cohort, N ​= ​53		
	Stable IAs, N ​= ​149	Unstable IAs, N ​= ​64	P value	Stable IAs, N ​= ​35	Unstable IAs, N ​= ​18	P value
CRstalk	0.55 ​± ​0.17	0.72 ​± ​0.22	<0.001^a^	0.60 ​± ​0.20	0.66 ​± ​0.14	0.176
AWE grade			<0.001^a^			0.323
Grade 0	37 (24.8)	2 (3.1)		10 (28.6)	2 (11.1)	
Grade 1	45 (30.2)	9 (14.1)		7 (20.0)	3 (16.7)	
Grade 2	61 (40.9)	45 (70.3)		15 (42.9)	9 (50.0)	
Grade 3	6 (4.0)	8 (12.5)		3 (8.6)	4 (22.2)	
Vmin (cm/s)	20.27 ​± ​11.8	15.87 ​± ​10.92	0.017^a^	22.86 ​± ​12.05	14.36 ​± ​12.64	0.005^a^
Vavg (cm/s)	33.49 ​± ​14.86	28.54 ​± ​13.26	0.026^a^	37.10 ​± ​16.44	27.56 ​± ​13.39	0.035^a^
Vmax (cm/s)	50.94 ​± ​21.61	42.43 ​± ​17.49	0.009^a^	55.19 ​± ​24.17	41.66 ​± ​18.30	0.059
Flowavg (ml/s)	1.53 ​± ​1.75	1.82 ​± ​1.68	0.048	1.59 ​± ​1.40	1.26 ​± ​0.99	0.425
Flowmax (ml/s)	2.69 ​± ​2.83	3.53 ​± ​3.30	0.012^a^	2.91 ​± ​2.38	2.33 ​± ​1.54	0.499
TAWSS (N/m2)	0.44 ​± ​0.32	0.22 ​± ​0.28	<0.001^a^	0.43 ​± ​0.32	0.28 ​± ​0.24	0.015^a^
WSSmax (N/m2)	0.76 ​± ​0.65	0.39 ​± ​0.40	<0.001^a^	0.78 ​± ​0.49	0.57 ​± ​0.36	0.058
PI	1.03 ​± ​0.58	1.02 ​± ​0.44	0.685	0.89 ​± ​0.29	1.07 ​± ​0.39	0.167

a Significant values. AR, aspect ratio; AWE, aneurysm wall enhancement; CRstalk, the aneurysm-to-pituitary stalk ratio; IA, intracranial aneurysm; MWR, major axis to width ratio; NSI, nonspherical index; PI, pulsatile index; TNR, transverse to neck ratio; UI, undulation index; V, velocity; VNR, volume-to-neck ratio; TAWSS, time-averaged wall shear stress.

### Accuracy comparisons of the different models for predicting aneurysm instability in the primary cohort

After feature selection, we constructed 7 different classification models. The performance of the models to predict aneurysm instability is summarized in Table 3, which revealed that the Hybrid Model (4D-Flow-LR and MicroAB-Net) achieved the best performance with an AUC value of 0.854 (95 ​% CI: 0.791, 0.916), followed by 4D-Flow-LR (AUC ​= ​0.809, 95 ​% CI: 0.740, 0.877), MicroAB-Net (AUC ​= ​0.807, 95 ​% CI: 0.738, 0.877), ELAPSS-LR (AUC ​= ​0.774, 95 ​% CI: 0.700, 0.848), AWE-LR (AUC ​= ​0.726, 95 ​% CI: 0.647, 0.804), PHASES-LR (AUC ​= ​0.725, 95 ​% CI: 0.646, 0.803), Radiomics (AUC ​= ​0.703, 95 ​% CI: 0.622, 0.783).

**Table 3:** Performances of the proposed hybrid model and baseline models in predicting aneurysm instability in the primary cohort.

Method	AUC	Accuracy	Sensitivity	Specificity	PPV	NPV
PHASES-LR	0.725 (0.646, 0.803)	0.662 [141/213] (0.596, 0.722)	0.750 [48/64] (0.630, 0.840)	0.624 [93/149] (0.544, 0.698)	0.462 [48/104] (0.369, 0.557)	0.853 [93/109] (0.773, 0.908)
ELAPSS-LR	0.774 (0.700, 0.848)	0.737 [157/213] (0.674, 0.792)	0.828 [53/64] (0.715, 0.902)	0.698 [104/149] (0.620, 0.766)	0.541 [53/98] (0.442, 0.636)	0.904 [104/115] (0.835, 0.947)
AWE-LR	0.726 (0.647, 0.804)	0.662 [141/213] (0.596, 0.722)	0.828 [53/64] (0.715, 0.902)	0.591 [88/149] (0.510, 0.666)	0.465 [53/114] (0.376, 0.556)	0.889 [88/99] (0.810, 0.938)
Radiomics	0.703 (0.622, 0.783)	0.620 [132/213] (0.553, 0.682)	0.812 [52/64] (0.698, 0.890)	0.537 [80/149] (0.457, 0.615)	0.430 [52/121] (0.345, 0.519)	0.870 [80/92] (0.784, 0.925)
MicroAB-Net	0.807 (0.738,0.877)	0.761 [162/213] (0.699, 0.813)	0.750 [48/64] (0.630, 0.840)	0.765 [114/149] (0.690, 0.826)	0.578 [48/83] (0.471, 0.679)	0.877 [114/130] (0.808, 0.923)
4D-Flow-LR	0.809 (0.740, 0.877)	0.784 [167/213] (0.724, 0.834)	0.682 [45/66] (0.561, 0.781)	0.830 [122/147] (0.760, 0.882)	0.643 [45/70] (0.525, 0.745)	0.853 [122/143] (0.785, 0.902)
Hybrid Model	0.854 (0.791, 0.916)	0.817 [174/213] (0.759, 0.863)	0.734 [47/64] (0.614, 0.827)	0.852 [127/149] (0.786, 0.901)	0.681 [47/69] (0.563, 0.779)	0.882 [127/144] (0.818, 0.926)

Data in parentheses are 95 ​% confidence intervals. AUC, area under curve; AWE, aneurysm wall enhancement; LR, logistic regression; MicroAB-Net, Multi-crop Attention Branch Network model; NPV, ​negative predictive value; PPV, ​positive predictive value.

**Fig. 3:**
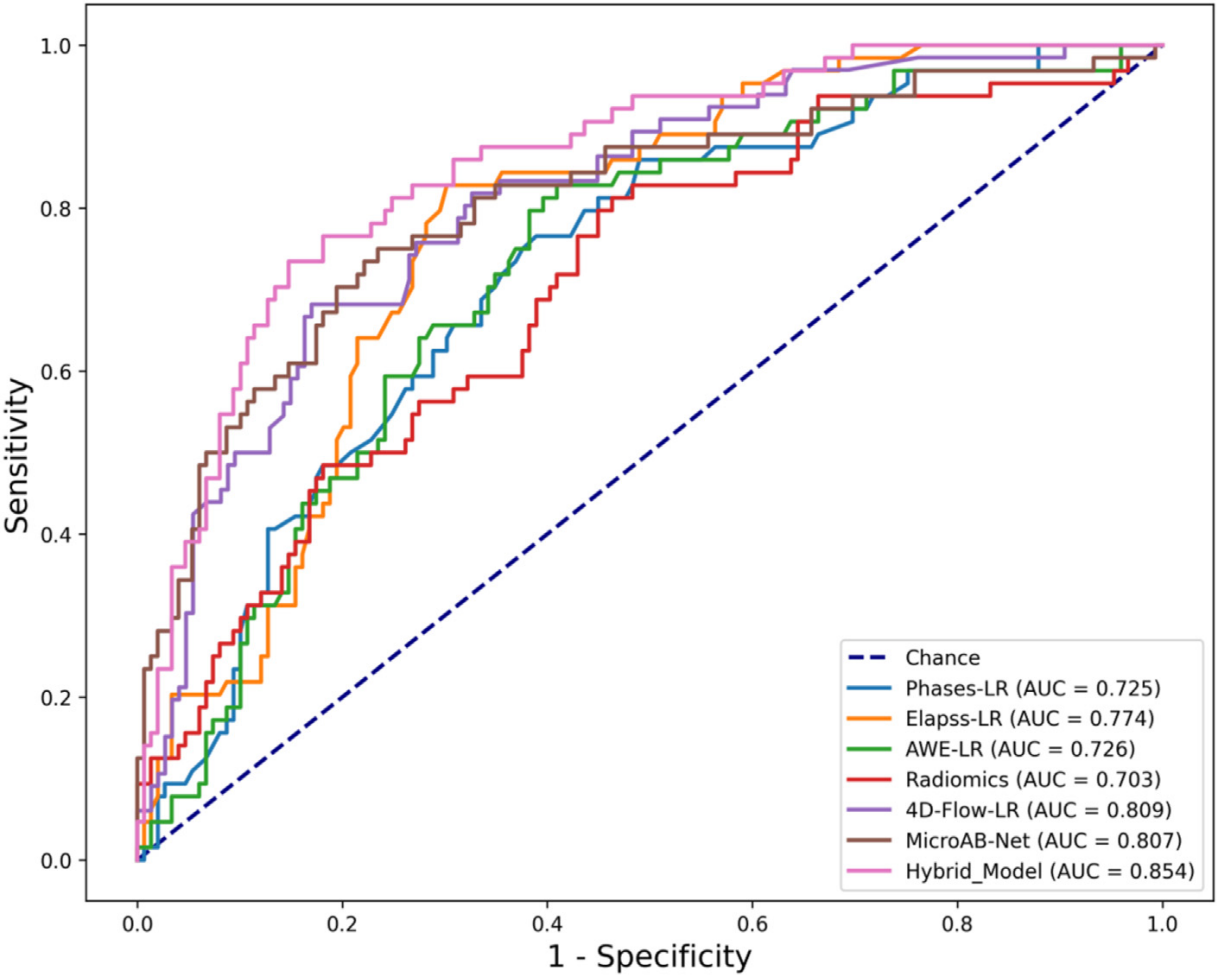
**ROC curves of seven prediction models in the primary cohort.** The Hybrid Model achieved the best performance to predict aneurysm instability than other prediction models with an AUC of 0.854. AUC, area under the curve; AWE, aneurysm wall enhancement; LR, logistic regression; MicroAB-Net, Multi-crop Attention Branch Network model; ROC, receiver operating characteristic.

**Fig. 4:**
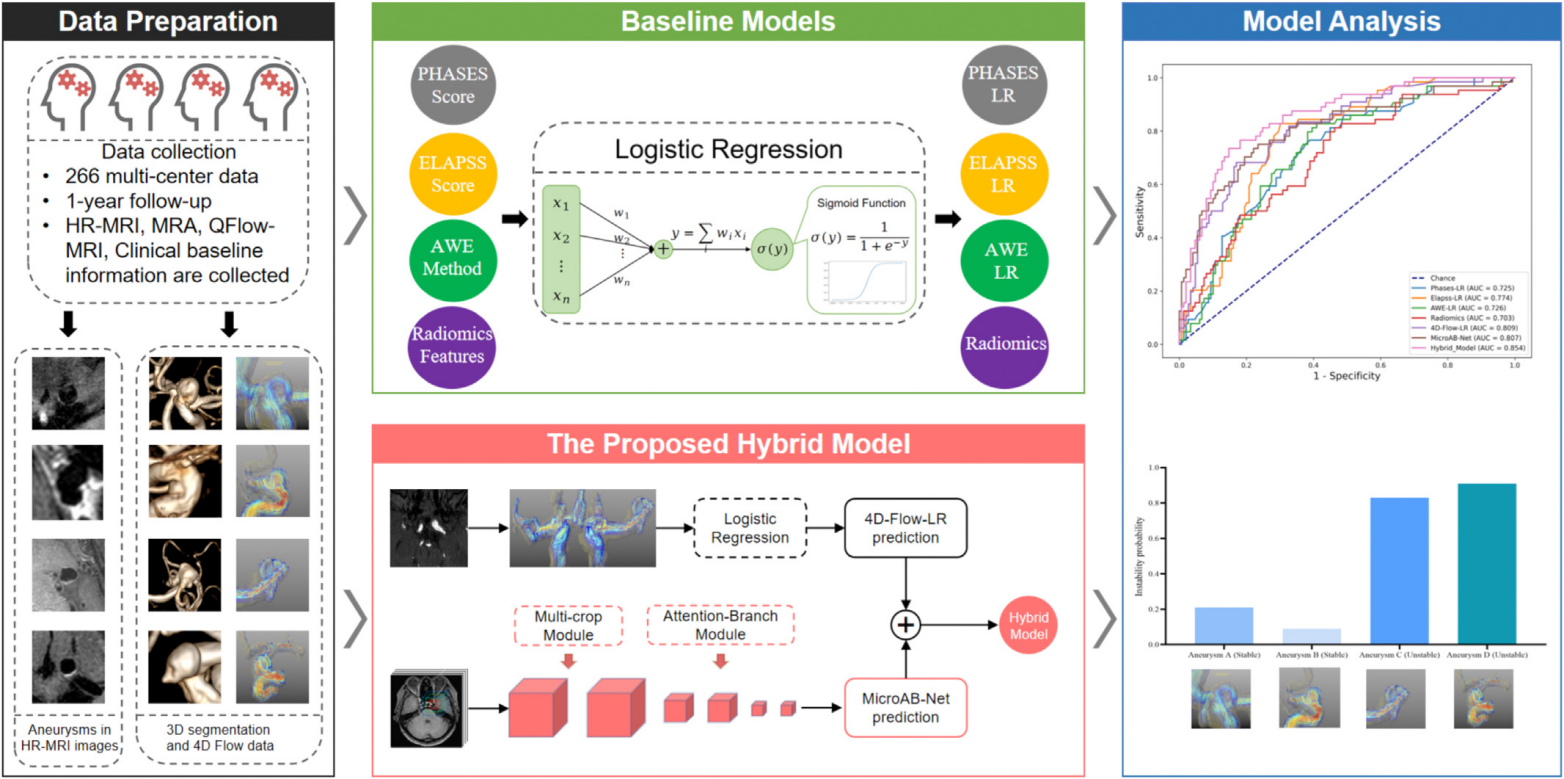
Case examples of the IA instability prediction flow chart by the proposed hybrid model.

To further analyze the differences between the models, we performed a DeLong's test and found that the Hybrid Model generally provided significant improvements over baseline models (p ​< ​0.05, Table 4). To create a visual comparison of the performance of the different models for predicting the risk of aneurysm rupture, we plotted the mean ROC curves from a ten-fold cross-validation experiment (Fig. 3). Case examples of the IA instability prediction procedure by the proposed hybrid model are shown in Fig. 4.

**Table 4:** The pair-wised P values of all models by DeLong's test in the primary cohort.

	PHASES-LR	ELAPSS-LR	AWE-LR	Radiomics	MicroAB-Net	4D-Flow-LR	Hybrid Model
PHASES-LR	–	0.106	0.986	0.631	0.039	0.071	<0.001
ELAPSS-LR	–	–	0.234	0.123	0.39	0.538	0.021
AWE-LR	–	–	–	0.572	0.037	0.081	<0.001
Radiomics	–	–	–	–	0.008	0.044	<0.001
MicroAB-Net	–	–	–	–	–	0.845	0.052
4D-Flow-LR	–	–	–	–	–	–	0.059
Hybrid Model	–	–	–	–	–	–	–

Data in parentheses are 95 ​% confidence intervals. Boldface type indicates statistical significance. AUC, area under curve; AWE, aneurysm wall enhancement; LR, logistic regression; MicroAB-Net, Multi-crop Attention Branch Network model.

### Validation of the different models for predicting aneurysm instability

In the validation cohort, forty-eight patients (35 women [72.9 ​%]; aged 55.0 years ​± ​10.77) with 53 IAs were included. In the validation cohort, 18 IAs presented with symptoms or morphological change or ruptured during follow-up periods. Therefore, 18 IAs were classified as unstable, while 35 IAs were stable. The median aneurysm size for stable IAs was 6.20 ​± ​2.12 for stable IAs and 8.80 ​± ​2.64 for unstable IAs (p ​< ​0.001). The demographics and clinical features and aneurysm characteristics are summarized in Table 1, Table 2. Parameters of interest in the primary cohort and the validation cohort are listed in Table S3.

All of the models were further tested in the validation cohort to assess their applicability and generalizability. The performance of the models is summarized in Table S4. The DeLong's test was also performed to analyze the differences between the Hybrid Model and other models (Table S5). These results indicated that the Hybrid model also provided significant improvements over almost all of the previous models with an AUC of 0.876 (95 ​% CI: 0.769,0.983).

### Outcome during 2-year follow-up in the overall cohort

During the 2-year follow-up period of the overall cohort, 154 patients with 174 IAs underwent surgical or interventional treatment. Among those without any surgical or interventional treatments, twenty-five aneurysms grew or ruptured. One of the ruptured aneurysms has been reported previously [20]. Specifically, to demonstrate the application of the aneurysm instability prediction workflow in clinical practice, one typical case of an aneurysm ruptured during follow-up is provided in Fig. S5.

## Discussion

IA instability may primarily involve interactions between the aneurysm wall and intra-aneurysm blood flow [8], which can be demonstrated through HR-MRI and 4D-Flow MRI, respectively. In this study, we developed a novel ensemble learning hybrid model by combining a 4D-Flow logistic regression model with an HR-MRI deep learning model named MicroAB-Net. To the best of our knowledge, we are the first to show that: 1) a hybrid model using 4D-Flow data and HR-MRI achieves promising performance in prediction of IA instability both in the primary cohort (AUC, 0.854) and the validation cohort (AUC, 0.876); 2) such a hybrid model maybe a supportive and complementary tool to facilitate clinicians in decision-making on whether to choose preventive treatment or conservative treatment for unruptured intracranial aneurysms.

Our hybrid IA instability prediction model performed better than the baseline models, including PHASES score, ELAPSS score, aneurysm wall enhancement, and radiomics models. Recent studies reveal that AI techniques, especially deep learning, are rapidly becoming a reliable method for the management of aneurysms [19,28]. Ou et al. established an aneurysm-rupture-prediction deep learning model based on CTA, MRA, and DSA that used only limited labeled data [19]. However, previous studies have not employed deep learning to extract image features from HR-MRI, which contains the real information of the aneurysmal wall. In the current study, we propose a new 3D image classification network (MicroAB-Net) that incorporates features of both the aneurysmal wall and aneurysm morphology. It is worth mentioning that our proposed MicroAB-Net alone outperformed some well-known rupture prediction models like clinical, PHASES score, AWE, and radiomics. Benefiting from the deep residual network architecture, the MicroAB-Net has a powerful ability to learn features related to aneurysm stability directly from HR-MRI images. We also introduced a multi-crop input policy to incorporate both aneurysmal wall and aneurysm morphology features. We then further applied an attention branch module to select the spatial regions contributing more to the IA instability prediction. We present heatmaps of the regions that presented features used by our MicroAB-Net in Fig. S5. In these heatmaps, regions with a higher value are more important for the MicroAB-Net. The important areas lie mainly on the aneurysmal walls or inside the aneurysms, which indicates the effectiveness of our design.

Previous rupture prediction models like PHASES score and ELAPSS score only include the characteristics of patients and limited morphological parameters of IAs [5,6], which may limit the accuracy of individualized IA stability prediction. In fact, both aneurysmal wall inflammation and intra-aneurysmal hemodynamics play crucial roles in the pathogenesis of IA [[7], [8], [9]]. Notably, inflammation was reported to play a key role in aneurysm initiation, growth, and rupture [8]. Recently, AWE on HR-MRI, which correlates well with aneurysmal wall inflammation in pathological studies, has emerged as a new imaging biomarker for IA instability [29]. High AWE may reflect high aneurysmal wall permeability (caused by aneurysmal wall inflammation), which allows contrast agent passage. Roa et al. reported that CR_stalk_ was the optimal quantitative AWE parameter for predicting aneurysm instability, with a cut-off value of 0.60 [14]. We found a similar CR_stalk_ cut-off value of 0.635 in this study. However, we found AWE-LR model to be inferior to the HR-MRI-based deep learning model. We consider that the reason for this may be that the deep learning architecture (CNN) is highly efficient at learning the relationships between heterogeneous data [30], while the qualitative and quantitative features of AWE only demonstrate a subjective assessment or a single value of signal intensity summarized from the digital images.

Radiomics is another approach that extracts image features and analyzes them through a data-driven method [31]. Ou et al. found that radiomics analysis showed differences between ruptured and unruptured IAs [31]. Liu et al. investigated PyRadiomics-derived morphological characteristics for discriminating between stable and unstable IAs using machine learning, and found that compactness was the most important morphological predictor [11]. However, in this study, radiomics was inferior to both the deep learning model MicroAB-Net and the Hybrid Model. The radiomics model uses manually extracted features that are not specifically designed for analyzing IA instability, and which may not represent the true characteristics of the aneurysm. In contrast, our deep learning model can learn specific features related to IA instability directly from the HR-MRI images and instability labels. Furthermore, intra-aneurysmal hemodynamics features are not analyzed in the radiomics model, whereas our Hybrid Model takes advantage of multimodality features (both aneurysmal wall and intra-aneurysmal hemodynamics features) when evaluating IA instability.

As the driving force of inflammation, intra-aneurysmal hemodynamics also play a crucial role in the progression of IAs [8]. Considering that the optimal model in this study used both 4D-Flow data and HR-MRI, we suggest that hemodynamics may need to be combined with aneurysmal wall features in future studies, to ensure a comprehensive evaluation of IA instability. In the current study, other parameters like mural thrombus and some morphological parameters also reach statistical significance in predicting aneurysm instability. Mural thrombus, indicating vasa vasorum and mural inflammation, has been reported to be associated with aneurysm instability [32,33]. In the future, a more comprehensive model, which incorporates more modalities and parameters (clinical, morphological, etc.) of patients, aneurysms and the parent artery, should be carried out [34].

This study has several strengths. First, new imaging modalities such as 4D-Flow MRI and HR-MRI can reflect the features most closely associated with aneurysms (intra-aneurysm hemodynamics and aneurysmal wall) and have added the reliability and accuracy in IA instability prediction in the current study. However, there are still several limitations. First, the limited resolution of the current 4D flow can't analyze small aneurysms [12]. Therefore, in accordance with previous studies [9,21], we excluded IAs <4 ​mm, which may have resulted in the overall larger aneurysm size in this study and reduced the applicability of the model to small IAs. Second, the new technique of acquiring 4D-Flow MRI from IAs has not been widely applied in China, and the research centers performing it are very limited. Therefore, this study recruited a small number of participants. Third, although the classification models are sophisticated, and the performance is derived and validated rigorously, the features rely on new MRI techniques that are not widely performed and require expert neuroradiology assessment. This reduces the generalizability of the approach. Finally, since the study was conducted in China, the results cannot be generalized to other countries.

In this study, we presented a promising IA instability prediction model (the Hybrid Model) and demonstrated its effectiveness in 237 patients. The Hybrid Model, which combines a deep learning predictive framework based on HR-MRI and hemodynamics based on 4D-Flow MRI, showed significantly improved prediction performance over previous models. Consequently, this model may serve as a valuable and complementary tool to facilitate clinicians in decision-making on the surgical treatment for unruptured intracranial aneurysms, although further validation is warranted. Furthermore, future research on the prediction of IA stability should consider incorporating intra-aneurysmal hemodynamics and the enhancement of the aneurysmal wall as critical factors.

## Ethics approval and consent to participate

The current study was approved by the Beijing Tiantan Hospital Institutional Review Board (IRB approval number: KY 2018-086-03), and written informed consents were obtained from each subject.

## Funding

This current study was supported by the Postdoctoral Fellowship Program of CPSF (GZC20231746), Project supported by Beijing Postdoctoral Research Foundation, 10.13039/501100001809National Natural Science Foundation of China (No.82171290; 82201427; 81771233), 10.13039/501100004826Natural Science Foundation of Beijing Municipality (No. 7222050, L192013), 10.13039/501100009601Beijing Municipal Administration of Hospitals' Ascent Plan (DFL20190501), and Research and Promotion Program of Appropriate Techniques for Intervention of Chinese High-risk Stroke People (GN-2020R0007).

## Author Contributions

Fei Peng: substantial contributions to conception and design, acquisition of data, drafting or revising of the manuscript critically for important intellectual content.

Jiaxiang Xia: substantial contributions to conception and design, acquisition of data.

Fandong Zhang, substantial contributions to conception and design, analysis and interpretation of data.

Shiyu Lu, substantial contributions to conception and design, analysis and interpretation of data.

Hao Wang, analysis and interpretation of data.

Jiashu Li, acquisition of data.

Xinmin Liu, acquisition of data.

Jiahuan Guo, acquisition of data.

Yao Zhong, acquisition of data.

Yonghong Duan, acquisition of data, analysis and interpretation of data.

Binbin Sui, analysis and interpretation of data.

Chuyang Ye, analysis and interpretation of data.

Yi Ju, final manuscript approval for submission.

Shuai Kang, final manuscript approval for submission.

Yizhou Yu, final manuscript approval for submission.

Xin Feng, analysis and interpretation of data, final manuscript approval for submission and publication.

Xingquan Zhao, final manuscript approval for submission and publication.

Rui Li, final manuscript approval for submission and publication.

Aihua Liu, final manuscript approval for submission and publication.

## Consent to participate

Informed consent was obtained from all individual participants included in the study.

## Research registration unique identifying number (UIN)

Name of the registry: Application study in evaluating the risk of intracranial aneurysm rupture based on 4D Flow MRI hemodynamic.

Unique Identifying number or registration ID: Chinese Clinical Trial Registry, ChiCTR2000038440.

Hyperlink to your specific registration (must be publicly accessible and will be checked): https://www.chictr.org.cn/bin/project/edit?pid=61468.

## Declaration of competing interest

The authors declare that they have no known competing financial interests or personal relationships that could have appeared to influence the work reported in this paper.

## Glossary

4D-Flow MRI: four-dimensional flow magnetic resonance imaging
HR-MRI: high-resolution magnetic resonance imaging
IA: intracranial aneurysm
MicroAB-Net: Multi-crop Attention Branch Network model
MRA: magnetic resonance angiography
CTA: computed tomography angiography
DSA: digital subtraction angiography
AWE: aneurysm wall enhancement
CRstalk: contrast ratio of the signal intensity between the aneurysmal wall and the pituitary stalk
AI: artificial intelligence
AUC: area under the curve
PPV: positive predictive value
NPV: negative predictive value
CI: confidence intervals
WSS: wall shear stress
PI: pulsatility index
V: velocity
