# Supplementary material for: Intracranial aneurysm instability prediction model based on 4D-Flow MRI and HR-MRI
Source: Neurotherapeutics. 2024 Nov 30;22(1):e00505. doi: 10.1016/j.neurot.2024.e00505 (PMC11742858; doi:10.1016/j.neurot.2024.e00505)
Supplement: Multimedia component 1 [file mmc1.docx]

**Supplemental Materials**

**Intracranial aneurysm instability prediction model based on 4D-Flow MRI and HR-MRI**

Supplemental Methods

Table S1-5

Figure S1-5

**Supplemental Methods**

**MicroAB-Net details**

The detailed structure of the feature extraction network is described as follow. There are 23 convolution layers in all. The size of all convolution kernels is 3x3x3. The first layer is plain convolution layer. Then, four residual stages are stacked. These four stages consist of 2,3,4,2 residual blocks, respectively, in sequence. Each residual block contains two convolution layers and a skip connection. We use a max-pooling layer after the third stage and an average pooling layer after the fourth stage. Finally, the features after the average pooling layer are fed into a softmax layer for instability classification.

**Radiomics model details**

In the radiomics model, the open source Python library Pyradiomics is used to extract radiomics features of the 3D aneurysm regions. A total of 1906 radiomic features are extracted, which contain first-order statistics, shape (3D), gray-level co-occurrence matrix (GLCM), gray level size zone matrix (GLSZM), gray level run length matrix (GLRLM), gray level dependence matrix (GLDM) and Neighbouring Gray Tone Difference Matrix (NGTDM). Then, 92 radiomic features were selected by the Joint Hypothesis Test to mitigate model overfitting. In detail, if the linear correlation coefficient between any two features is greater than a given threshold (here we use 0.9), the one with less impact on the aneurysm instability classification will be discarded. Last, a logistic regression classification model is performed to map the selected features into aneurysm stability score. In addition, we use L1 regularization during logistic regression to further mitigate overfitting.

**Supplemental Tables**

**Table S1. Summary of all parameters included in each model**

| **Categories of the parameters** | **Parameters** |
| --- | --- |
| PHASES score | population, hypertension, age, size, earlier rupture, and site |
| ELAPSS score | earlier subarachnoid hemorrhage, location, age, population, size, and shape |
| Aneurysm wall enhancement | AWE grades (0/1/2/3), CR_stalk_ |
| Hemodynamic | V_min_, V_avg_, V_max_, Flow_avg_, Flow_max_, TAWSS, WSS_max_ |

AWE, aneurysmal wall enhancement; CR_stalk_, the aneurysm-to-pituitary stalk ratio; SAH, subarachnoid hemorrhage; V, velocity; TAWSS, time-averaged wall shear stress.

**Table S2. Inter-observer agreement analyses in the primary cohort**

| Characteristics | Reader 1 | Reader 2 | Kappa or ICC (95% CI) |
| --- | --- | --- | --- |
| Size | 8.47 ± 5.05 | 9.70 ± 5.46 | 0.893 (0.774-0.940) |
| TAWSS | 0.37 ± 0.33 | 0.39 ± 0.25 | 0.877 (0.809-0.917) |
| AWE grades (0/1/2/3) | 39/54/106/14 | 38/57/102/16 | 0.914 (0.867-0.961) |
| CR_stalk_ | 0.60 ± 0.21 | 0.64 ± 0.25 | 0.881 (0.88-0.922) |

AWE, aneurysm wall enhancement; CI, confidence interval; CR_stalk_, the aneurysm-to-pituitary stalk ratio; ICC, intraclass correlation coefficient; TAWSS, time-averaged wall shear stress.

**Table S3. Comparisons of demographic, clinical, morphological, hemodynamic, and aneurysm wall enhancement features between the primary cohort and validation cohort**

| **Variable** | **Primary cohort, N = 213** | **Validation cohort, N = 53** |
| --- | --- | --- |
| Age | 58.90 ± 10.32 | 55.0 ± 10.77 |
| Female | 148 | 35 |
| Hypertension | 142 | 27 |
| Diabetes | 22 | 8 |
| Dislipidemia | 80 | 15 |
| Coronary artery disease | 24 | 3 |
| Smoking history | 55 | 14 |
| Aneurysm location |  |  |
| Anterior circulation | 159 | 34 |
| Posterior circulation | 54 | 19 |
| Side-wall | 143 | 40 |
| Mural thrombus | 65 | 9 |
| Size | 8.47 ± 5.05 | 7.27 ± 2.66 |
| CR_stalk_ | 0.60 ± 0.21 | 0.53 ± 0.18 |
| AWE grade |  |  |
| Grade 0 | 39 | 14 |
| Grade 1 | 54 | 15 |
| Grade 2 | 106 | 19 |
| Grade 3 | 14 | 5 |
| V_min_ | 18.95 ± 11.75 | 21.48 ± 11.90 |
| V_avg_ | 32.00 ± 14.55 | 34.50 ± 14.80 |
| V_max_ | 48.38 ± 20.80 | 51.77 ± 21.41 |
| Flow_avg_ | 1.61 ± 1.73 | 1.54 ± 1.57 |
| Flow_max_ | 2.94 ± 3.00 | 2.73 ± 2.58 |
| TAWSS | 0.37 ± 0.33 | 0.36 ± 0.27 |
| WSS_max_ | 0.65 ± 0.61 | 0.72 ± 0.87 |
| PI | 1.03 ± 0.54 | 0.94 ± 0.33 |
| Phases Score | 6.21 ± 3.69 | 6.70 ± 4.00 |
| Elapss Score | 19.10 ± 7.25 | 21.62 ± 7.18 |

*Significant values. AR, aspect ratio; AWE, aneurysm wall enhancement; CR_stalk_, the aneurysm-to-pituitary stalk ratio; IA, intracranial aneurysm; MWR, major axis to width ratio; NSI, nonspherical index; PI, pulsatile index; TNR, transverse to neck ratio; UI, undulation index; V, velocity; VNR, volume-to-neck ratio; TAWSS, time-averaged wall shear stress.

**Table S4. Performances of the proposed hybrid model and baseline models in predicting aneurysm instability in the validation cohort.**

| Method | AUC | Accuracy | Sensitivity | Specificity | PPV | NPV |
| --- | --- | --- | --- | --- | --- | --- |
| PHASES-LR | 0.739 (0.595,0.884) | 0.698[37/53]  (0.564, 0.805) | 0.950[19/20] (0.743, 1.007) | 0.545[18/33] (0.380, 0.701) | 0.559[19/34] (0.395, 0.711) | 0.947[18/19] (0.732, 1.007) |
| ELAPSS-LR | 0.760 (0.620,0.900) | 0.717[38/53], (0.583, 0.821) | 0.950[19/20] (0.743, 1.007) | 0.576[19/33] (0.408, 0.727) | 0.576[19/33] (0.408, 0.727) | 0.950[19/20] (0.743, 1.007) |
| AWE-LR | 0.717 (0.569,0.865) | 0.698[37/53]  (0.564, 0.805) | 0.850[17/20] (0.629, 0.954) | 0.606[20/33] (0.436, 0.753) | 0.567[17/30] (0.392, 0.726) | 0.870[20/23] (0.668, 0.961) |
| Radiomics | 0.474 (0.313,0.635) | 0.566[30/53]  (0.433, 0.690) | 0.450[9/20] (0.259, 0.658) | 0.636[21/33] (0.465, 0.778) | 0.429[9/21] (0.245, 0.635) | 0.656[21/32] (0.482, 0.796) |
| MicroAB-Net | 0.809 (0.681,0.938) | 0.792[42/53] (0.663, 0.881) | 0.800[16/20] (0.577, 0.923) | 0.788[26/33] (0.619, 0.895) | 0.696[16/23] (0.489, 0.844) | 0.867[26/30] (0.695, 0.952) |
| 4D-Flow-LR | 0.858 (0.744,0.971) | 0.811[43/53] (0.684, 0.895) | 0.800[16/20] (0.577, 0.923) | 0.818[27/33] (0.651, 0.916) | 0.727[16/22] (0.515, 0.870) | 0.871[27/31] (0.704, 0.953) |
| Hybrid Model | 0.876 (0.769,0.983) | 0.736[39/53] (0.603, 0.836) | 1.000[20/20] (0.806, 1.027) | 0.576[19/33]  (0.408, 0.727) | 0.588[20/34] (0.422, 0.736) | 1.000[19/19] (0.798, 1.028) |

Data in parentheses are 95% confidence intervals. AUC, area under curve; AWE, aneurysm wall enhancement; LR, logistic regression; MicroAB-Net, Multi-crop Attention Branch Network model; NPV, negative predictive value; PPV, positive predictive value.

**Table S5.** **P values for the differences between the Hybrid Model and previous models by DeLong's test in the validation cohort.**

|  | Clinical-LR | Phases-LR | Elapss-LR | AWE-LR | Morpholoy-LR | Radiomics | 3D-Resnet |
| --- | --- | --- | --- | --- | --- | --- | --- |
| Hybrid Model | **0.001** | **0.040** | 0.134 | **0.018** | **0.015** | **0.000** | **0.031** |

Data in parentheses are 95% confidence intervals. Boldface type indicates statistical significance. AUC, area under curve; AWE, aneurysm wall enhancement; LR, logistic regression; MicroAB-Net, Multi-crop Attention Branch Network model.

**Supplemental Figures**


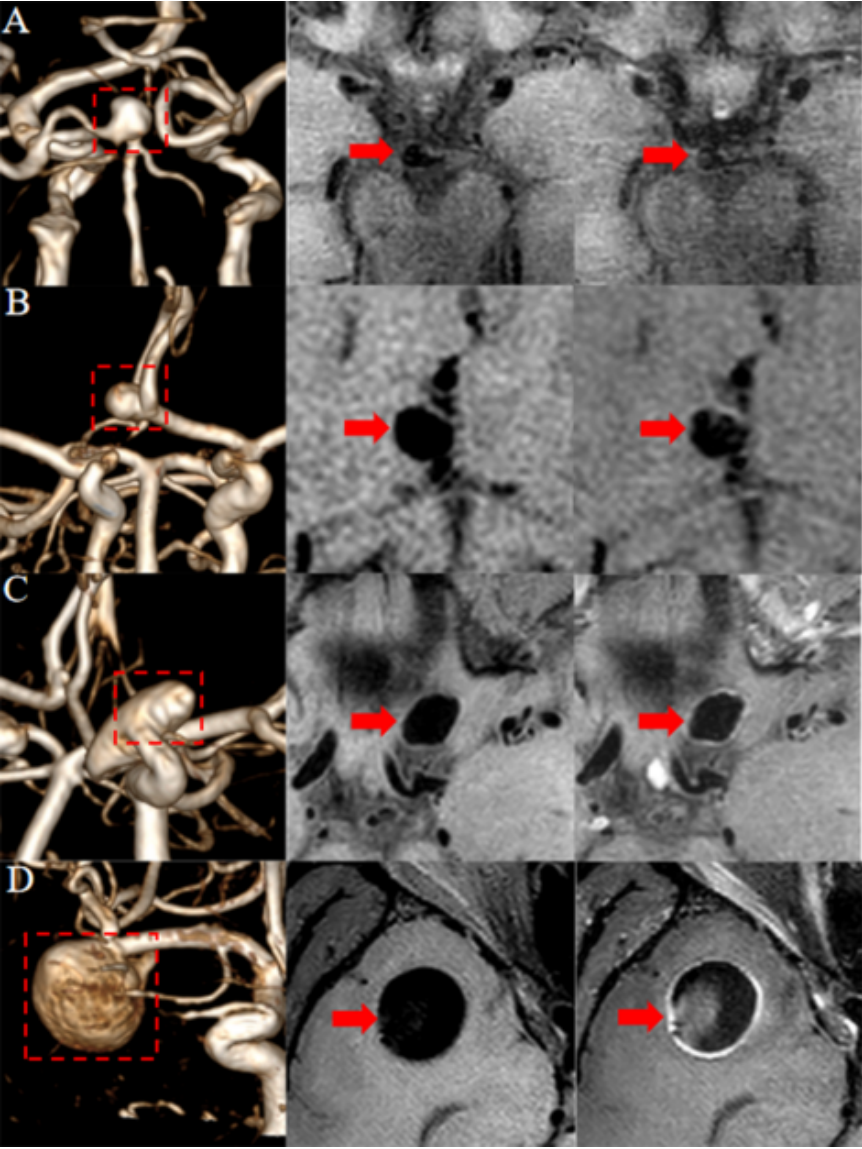


**Figure S1. Representative cases of aneurysm wall enhancement demonstrated by four grades.**

Each aneurysm is presented on a row. The left column shows the time-of-flight MR angiography, the middle column shows the pre-contrast vessel wall images and the right column shows the post-contrast vessel wall images. A, Grade 0: nonenhancing basilar tip aneurysm; B, grade 1: focal thick wall enhancement of a anterior communicating artery aneurysm; C, grade 2: circumferential wall enhancement of a internal carotid artery aneurysm; and D, grade 3: thick (> 1 mm) circumferential wall enhancement of a middle cerebral artery aneurysm.


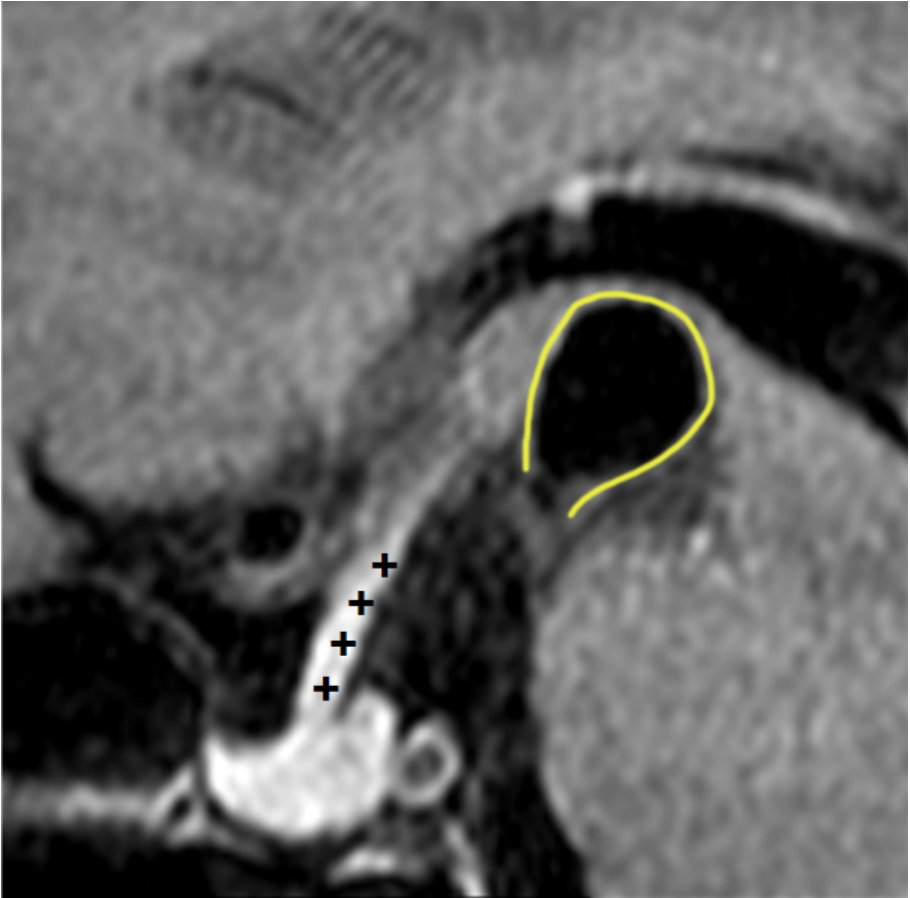


**Figure S2. Quantification of aneurysm wall enhancement.**

CR_stalk_ is calculated as the contrast ratio of the average signal intensity of the aneurysm wall (1166) to the average value of signal intensity of the four randomized points in the pituitary stalk (1564). Thus, CR_stalk_ is 0.75 in this case. CR_stalk_, the aneurysm-to-pituitary stalk ratio.

**
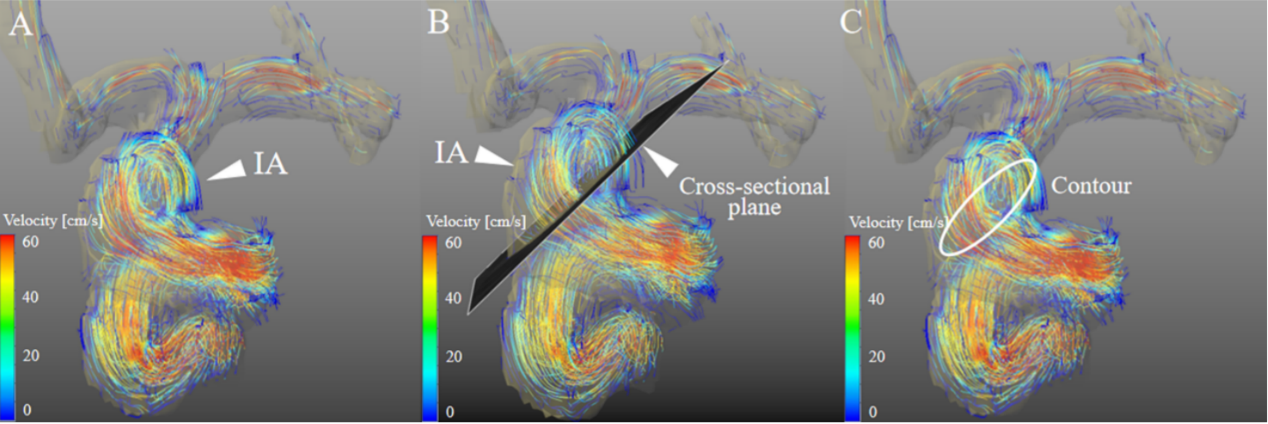
**

**Figure S3. Quantification of intra-aneurysmal hemodynamics.**

Flow pattern visualization of the intracranial aneurysm (IA) was performed by streamlines (A) and the largest cross-section of IA (B) were showed in IA. Hemodynamic measurements within the contour (C) was conducted in IA. IA, intracranial aneurysm.


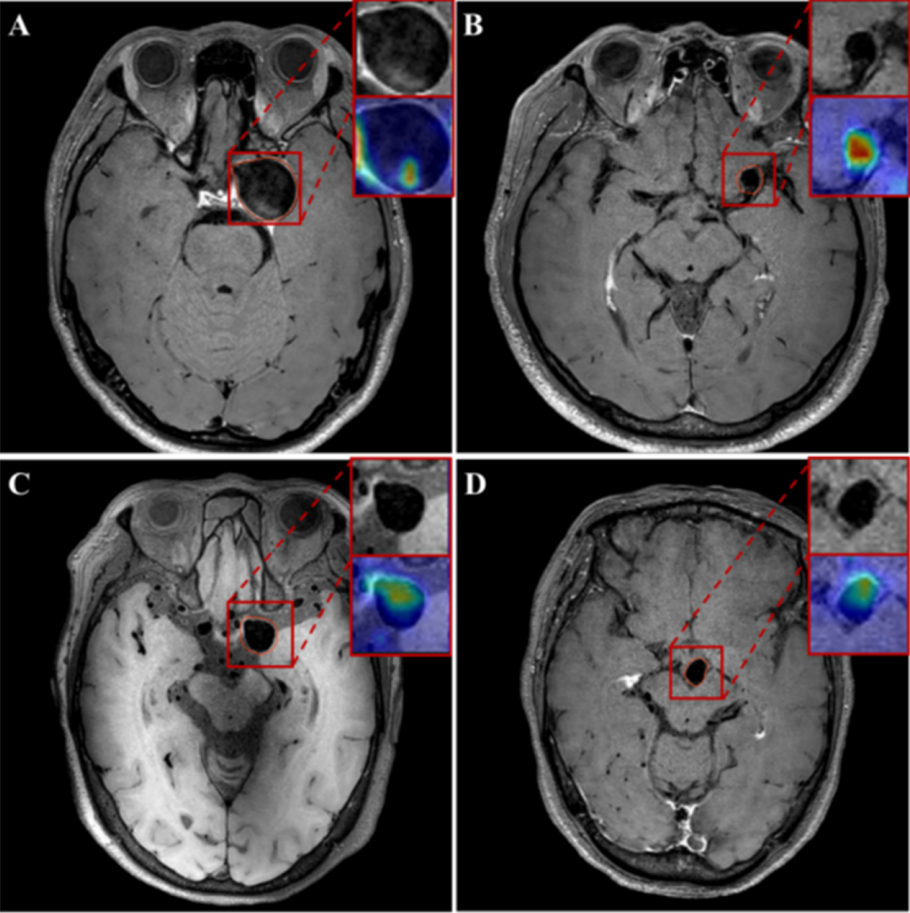


**Figure S4. Examples of the IA cases and the heatmaps learned by MicroAB-Net respectively.**

The case (A) and (B) are unstable IAs, while the case (C) and (D) are stable ones. For each case, the local image patch of the IA and the heatmap learned by MicroAB-Net are shown at the top right-hand corner. In the heatmaps, regions with higher value are more important for the MicroAB-Net. IA, intracranial aneurysm; MicroAB-Net, Multi-crop Attention Branch Network model.

**
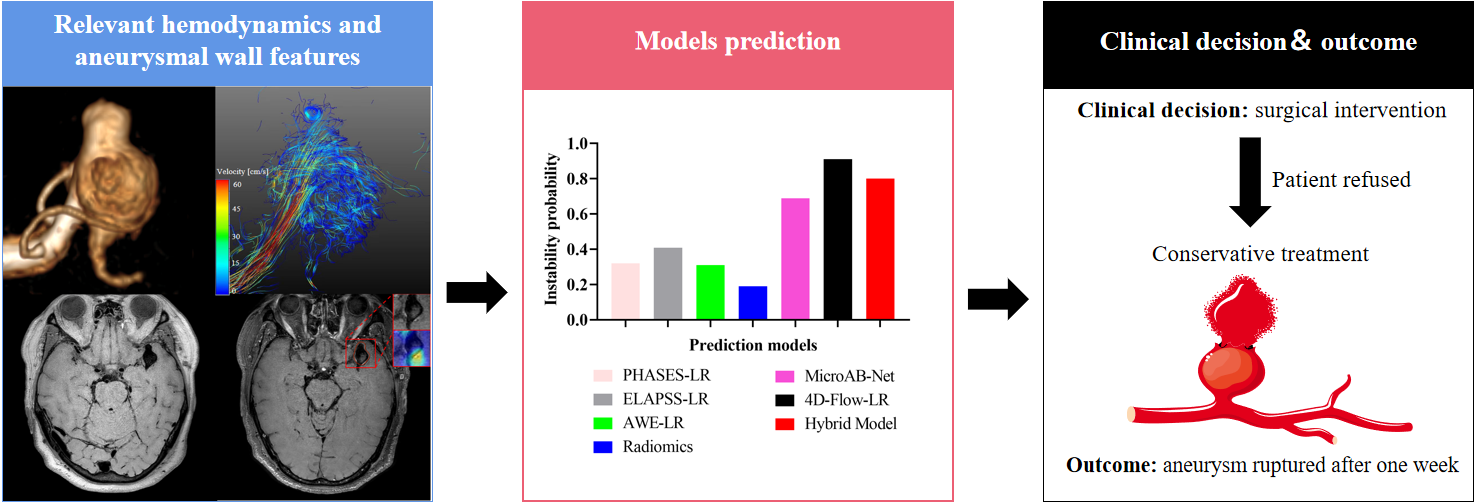
**

**Figure S5. One typical case of an aneurysm ruptured during follow-up.** An adult with a left middle cerebral artery bifurcation aneurysm was noted on magnetic resonance imaging. 4D-flow MRI revealed a concentrated jet flow impacting directly on the aneurysm daughter sac. Aneurysmal wall enhancement in HR-MRI was found, and the heatmap learned by MicroAB-Net also revealed the regions with high value. Seven models were performed to predict the probability of aneurysm instability. The Hybrid Model revealed a high probability of aneurysm instability (0.80). Then, clinician suggested surgical intervention, while the patient refused and chose conservative treatment. Finally, the aneurysm ruptured after one week when the patient was defecating in the morning. 4D-flow MRI, four-dimensional flow magnetic resonance imaging; HR-MRI, high-resolution magnetic resonance imaging; MicroAB-Net, Multi-crop Attention Branch Network model.
